# Open science and transparency are our strongest tools in the fight against fraudulent publishing activities

**DOI:** 10.1371/journal.pmed.1004774

**Published:** 2025-09-26

**Authors:** Helen Lumbard, Daniel Routledge

**Affiliations:** Public Library of Science, Cambridge, United Kingdom

## Abstract

PLOS Medicine Executive Editor, Helen Lumbard, and Senior Editor, Front Section, Daniel Routledge, call for the scientific community to tackle research fraud and paper mills head on by creating robust systems of data sharing and transparency.

## Teaser

Systematic fraud threatens the integrity of science, with paper and review mills distorting the evidence base in medicine and global health. Data transparency—once seen mainly as a driver of discovery—must now be recognized as a frontline defense against misconduct. Only through open data and coordinated action can we safeguard trust in research and its impact on health.

Scientific progress depends on trust that research findings are robust, reproducible, and honestly reported. In recent years, this trust has been increasingly threatened by the rise of systematic research fraud, including paper mills, review mills, and other coordinated attempts to manipulate the scholarly record. In this context, data transparency must be reframed not only as a scientific virtue, but as a frontline defense against research misconduct.

Data sharing has long been advocated to allow replication, accelerate discovery, and foster collaboration [1]. Yet, as the academic publishing ecosystem confronts new and evolving threats, it is becoming clear that data transparency also plays a critical role in detecting fraudulent or fabricated research [2]. Shared data can expose inconsistencies, reveal implausible patterns, and empower both journals and third-party watchdogs to conduct deeper investigations.

Paper mills—services that produce and sell fraudulent manuscripts—often operate at scale and target journals across disciplines, including those in clinical and global health fields. These papers may present fabricated data, recycled content, and/or attempt to manipulate peer review processes [3]. In recent years, publishers have seen growing numbers of paper mill submissions; it is estimated that in 2022, 1.5%–2% of scientific papers came from paper mills, with this number increasing to 3% in medicine and biology [4]. Meanwhile, review mills compromise editorial integrity by infiltrating peer review systems, often with fake reviewer identities, inauthentic favorable reviews, and/or coordinated citation practices [5]. The consequences of these actions are not superficial; they distort the scientific record and could ultimately misinform clinical practice, public health policy, and funding priorities.

In clinical research, false data can lead to incorrect treatment recommendations, patient harm, and loss of trust in evidence-based medicine. In global health, where research often informs urgent responses to health crises, guides allocation of limited resources, and influences national and international health policies, the risks are magnified. Fraudulent studies in these areas not only undermine science but can also cause direct damage to vulnerable populations and health systems already under strain [6,7].

*PLOS Medicine*, like many journals and publishers, has taken significant steps to detect and address these threats, including enhanced pre- and post-publication checks. These efforts are strengthened through collaboration with initiatives such as United2Act, which coordinates action against paper mill activities, alongside growing research into this malpractice, and increased awareness among editors, reviewers, and readers [8–10]. Together with the development of more sophisticated detection tools, this heightened vigilance has already led to the identification and retraction of thousands of fraudulent articles across multiple publishers [11,12]. Yet, as in medicine, prevention remains the best treatment: while retrospective action is laudable, earlier detection and deterrence must be our focus. However, editorial detection can only go so far without institutional support, coordinated action, and, critically, transparent and accessible data.

While legitimate barriers to data sharing exist—particularly concerning patient privacy, national regulations, and ethical safeguards—blanket refusals to share data, or vague statements that data are “available on request,” do not meet the threshold for good practice. Increasingly, such statements raise red flags, especially in studies with statistical anomalies, improbable sample sizes, or poorly described methods. At *PLOS Medicine*, we have mandated since 2014 that authors make all data needed to replicate their study’s findings publicly available at the time of publication, without restriction.

This ensures transparency, reproducibility, and the widest possible benefit from the research. If there are legitimate legal or ethical reasons why a dataset cannot be shared openly, authors must explain these restrictions clearly and indicate how others can request access to the data in a responsible way.

In addition to promoting data availability and transparent reporting, we (the scientific community) must act in unity to protect the scholarly record and the real-world systems it informs.

We call on:

- Authors to prioritize data availability and to plan for open data from the outset of research.
- Funders and institutions to invest in ethical, secure infrastructure for data sharing—especially in under-resourced settings—and require a robust data management plan to ensure transparency, reproducibility, and responsible stewardship of research data.
- Journals and publishers to adopt robust data policies, support editors in investigations, and share intelligence about suspected fraud.
- Peer reviewers to scrutinize data availability statements and question inconsistencies.
- Readers and the wider research community to remain vigilant and engaged in post-publication review, open commentary, and data reanalysis.

We also urge collaborative action across the publishing industry, clinicians, medical researchers, and global health stakeholders to develop shared standards, tools, and coordinated responses to detect and deter paper and review mills. No single journal, institution, or sector can tackle this challenge alone.

Transparency is not a panacea, but it is a powerful tool. By making data more open and research processes more visible, we strengthen the foundations of trustworthy, evidence-based science. In doing so, we safeguard the integrity of clinical and global health research and uphold our shared responsibility to the people and communities that research ultimately aims to serve.
